# The Role of Demographics, Social Deprivation and Ethnicity on Anal Squamous Cell Carcinoma Incidence in England

**DOI:** 10.3390/jcm10163621

**Published:** 2021-08-17

**Authors:** Danielle R. L. Brogden, Christos Kontovounisios, Sundhiya Mandalia, Paris Tekkis, Sarah C. Mills

**Affiliations:** 1 Chelsea and Westminster Hospitals NHS Foundation Trust, London SW10 9NH, UK; daniellebrowning@nhs.net (D.R.L.B.); p.tekkis@imperial.ac.uk (P.T.); sarah.vonroon@gmail.com (S.C.M.); 2 Department of Surgery and Cancer, Imperial College London, London SW10 9NH, UK; s.mandalia@imperial.ac.uk; 3 Royal Marsden NHS Foundation Trust, London SW3 6JJ, UK

**Keywords:** Anal Squamous Cell Carcinoma, HIV, chemoradiotherapy, ethnicity, socioeconomic status, HPV

## Abstract

Anal Squamous Cell Carcinoma (ASCC) is an HPV-related malignancy with increasing incidence in high-income economies. Although ethnicity and social deprivation are known to be risk factors in other malignancies, little is known about socioeconomic status and risk of ASCC. This is a cross-sectional study following the STROBE Statement. Demographic data from the English Clinical Outcomes and Services Dataset (COSD) were extracted for all patients diagnosed with ASCC in England between 2013 and 2018. Outcomes included ethnicity, social deprivation, staging and treatment. This study included 5457 patients. Incidence increased by 23.4% in 5 years, with female incidence increasing more rapidly than male incidence (28.6% vs. 13.5%). Men were more likely to present with early staging (*p* < 0.001) and have surgery as their only treatment (*p* < 0.001). The rate of incidence of Stage 1 tumours in men was 106.9%; however, women had the greatest increase in metastatic tumours (76.1%). Black Caribbean and Black African patients were more likely to present at an earlier age with later staging (*p* < 0.001) and social deprivation was associated with younger age (*p* < 0.001). ASCC incidence is rapidly increasing in patterns consistent with two separate populations: one male with early staging, the other female and related to social deprivation and ethnicity factors.

## 1. Introduction

Anal Squamous Cell Carcinoma (ASCC) is an uncommon Human Papillomavirus (HPV) related cancer with an incidence of 1–2 cases per 100,000 people [1]. It has a known dysplastic precursor; Anal Intraepithelial Neoplasia (AIN), which usually acts similarly to Cervical Intraepithelial Neoplasia (CIN), progressing from low grade to high grade dysplasia with persistent HPV infection until an invasive malignancy develops.

The incidence rates of ASCC in high-income countries are rapidly rising [2] and in particular, in the United Kingdom (UK). Islami et al. 2017 states that the annual increase of ASCC incidence between 1979–2007 is 3.3% in men (95% CI 2.7–3.8) and 4.5% in women (95% CI 3.9–5.1) [2].

Although it is widely accepted that socioeconomic deprivation has an impact on the incidence [3] and prognosis [4,5,6] of many adult tumours, including other more common HPV related cancers such as cervical and head and neck tumours [7]. Due to its relative rarity, there is little in the worldwide literature about the role of ethnicity and social deprivation in the incidence and prognosis of ASCC. Three papers were identified; Celie et al. (2017) used the American Surveillance, Epidemiology and End Results (SEER) database to examine the relationship between socioeconomic status and ASCC outcomes and stage in patients diagnosed from 1988–2011. They identified that women and patients with a low socioeconomic status (SES) were more likely to present with advanced staging, receive radiotherapy and have lower survival rates than male patients or patients with high SES [8]. Nevertheless, Lin et al. (2018), using the same database for patients diagnosed from 2004–2013, agreed that patients living in geographical areas with low median household income had poorer survival; however, their data suggested that male patients (rather than female patients), African American patients and older age were associated with worse survival [9]. A further American study looking at the relationship of SES and end outcomes in patients diagnosed with ASCC suggested that low SES was not associated with stage but with a reduction in relapse free and overall survival [10].

It is unclear if there are differences in demographics and SES in ASCC in populations with universal healthcare such as the United Kingdom. We therefore examined the data available to investigate the demographics and SES of patients diagnosed with ASCC between 2013 and 2018 in England.

## 2. Materials and Methods

This is a cross-sectional study following the “strengthening the reporting of observational studies in epidemiology (STROBE)” statement [11]. Patients diagnosed with ASCC in England between January 2013 and June 2018 were identified using the Cancer Outcomes and Services Dataset (COSD). COSD is an anonymised Public Health England maintained dataset that includes demographic data as well as staging and treatment received stratified by NHS hospital and Clinical Commissioning Group (CCG).

Data extracted from COSD included gender, age, staging, ethnicity, treatment, and deprivation score. Age at diagnosis was classified into 5-year intervals. Deprivation was stratified into population quintiles as described by the UK Government English Indices of Deprivation where “Score 1” represents 20% of the population with the highest SES and “Score 5” represents 20% of the population with the lowest SES [12]. Treatment received included categorical data (yes/no) on whether patients received chemotherapy, radiotherapy and surgery for ASCC.

Data were also extracted from the Office of National Statistics (ONS) public bulletins that describe demographic data within CCGs.

### 2.1. Study Outcomes

The primary end outcome was ASCC incidence from January 2013–December 2017, which was calculated using the ONS data. Secondary outcomes were the relationship between cancer incidence and staging, gender, ethnicity and SES.

### 2.2. Inclusion Criteria

All patients over the age of 18 years with ASCC were included.

### 2.3. Exclusion Criteria

Patients under the age of 18 years and patients with histology other than ASCC were excluded.

### 2.4. Data Collection

Data were extracted by one author and collated by CCG to be checked for accuracy and duplication.

### 2.5. Statistical Analysis

Data were analysed using SPSS Statistics software. All outcomes extracted from COSD were expressed in categorical fields; therefore, comparisons of outcomes from COSD were analysed using Chi squared or Fisher’s Exact Tests. A statistically significant *p* value for this analysis was defined as *p* < 0.05. Incidence was calculated per 100,000 people in England per year. Any missing data fields were classified as “unknown” within the CODS database. Missing data were not excluded from the analysis and “unknown” variables are included with tables and figures to prevent bias.

## 3. Results

5457 patients were diagnosed with ASCC between January 2013 and June 2018. 3692 were female (67.7%), 12.5% were classified as Stage 1, 24.8% were classified as Stage 2, 36.0% were classified as Stage 3 and 7.5% were classified as Stage 4. The demographics of the population stratified by gender are detailed in Table 1. There was no difference in age, ethnicity and deprivation score between both genders; however, male patients were more likely to have early staging (Stage 1 and 2: 40.0% vs. 36.1%, *p* < 0.001).

### 3.1. Ethnicity

93.2% of the population were “Caucasian”, 1.2% identified as “Black”, 0.1% “Chinese”, 0.7% “Indian Subcontinent”, 0.4% “Mixed Ethnicity”, 1.0% “Other Ethnicity”, 0.2% “Other Asian” and 3.5% “Unknown Ethnicity”.

Although there was no effect of gender on ethnicity of patients diagnosed with ASCC, identifying as Black African or Black Caribbean was associated with presenting with Stage 3 or Stage 4 disease (*p* = 0.02) and younger age at diagnosis (54.8% < 55 years at diagnosis vs. 25.4% Caucasian < 55 years at diagnosis, *p* < 0.001).

Patients with ethnicities other than Caucasian were more likely to have higher levels of social deprivation (*p* < 0.001).

### 3.2. Social Deprivation

Social deprivation is divided into five quintiles using the UK Government English Indices of Deprivation [12]. Deprivation Score 1 represents 20% of the population with the least social deprivation and deprivation Score 5 represents the 20% of people with the highest levels of social deprivation in the population. Deprivation Score 1 was underrepresented in this dataset, only 15.9% of patients diagnosed with ASCC were classified as within the least deprived quintile in England. The rest of the quintiles are relatively equally spread between deprivation scores.

Younger age was associated with low SES in this cohort (Score 4 and 5; 52.7% of patients < 55 years, Score 1 and 2; 44.2% of patients < 55 years, *p* < 0.001); however, SES did not affect staging. Controlling for gender did not affect the statistical relationship between age, staging and ethnicity and SES.

### 3.3. Staging and Treatment

Patients were more likely to receive surgery as their only treatment if they were Stage 1 (*p* < 0.001) and more likely to receive radiotherapy alone (*p* = 0.01), surgery and chemotherapy (*p* < 0.001) and no treatment (*p* < 0.001) if they were Stage 4 at diagnosis (Table 2). Stage 3 had the highest patient numbers and was also associated with receiving surgery and chemoradiotherapy as a treatment (*p* < 0.001). Men were less likely to receive radiotherapy alone for early-stage tumours (*p* = 0.003) and more likely to have surgery alone (*p* < 0.001). Women were more likely to receive chemoradiotherapy (*p* < 0.001). Men with early node negative staging were more likely to receive surgery alone (*p* = 0.01), whereas women were more likely to receive chemoradiotherapy at Stage 2 (*p* = 0.03).

Patients diagnosed with Stage 1 tumours were more likely to be younger at diagnosis than patients diagnosed with late-stage tumours (*p* < 0.001) and younger early-stage patients were more likely to receive surgery as the only treatment for their malignancy (*p* = 0.02).

Receiving radiotherapy alone, no treatment, surgery with radiotherapy, chemoradiotherapy were all associated with increasing age of patient (*p* < 0.001). Meanwhile, receiving all three treatment modalities was associated with being between the ages of 40–75 years on diagnosis (*p* < 0.001).

Caucasian patients were more likely to receive chemoradiotherapy (37.7% vs. 25.0%, *p* = 0.01) and less likely to receive no treatment at all (15.1% vs. 28.1%, *p* < 0.001) when compared to Black African or Black Caribbean patients; this is due to Black African or Black Caribbean patients presenting with more advanced staging. However, Caucasian patients with Stage 1 disease were less likely to receive no treatment for their malignancy when compared with other ethnic groups (*p* = 0.01).

Increasing deprivation was associated with more patients receiving surgery and radiotherapy (*p* = 0.04) and less patients receiving chemoradiotherapy as a treatment modality (*p* = 0.02). At Stage 3, patients with lower levels of deprivation were more likely to receive chemoradiotherapy for their disease (*p* < 0.001, Chi Squared Test for trend), whereas deprived patients were more likely to receive surgery, chemotherapy and radiotherapy for their malignancy (*p* = 0.03, Chi Squared Test for trend).

## 4. Incidence of Anal Squamous Cell Carcinoma

In 2017, the incidence of ASCC in England for both genders was 1.9 per 100,000 people. This represented a 23.4% increase in 5 years.

**Figure 1:**
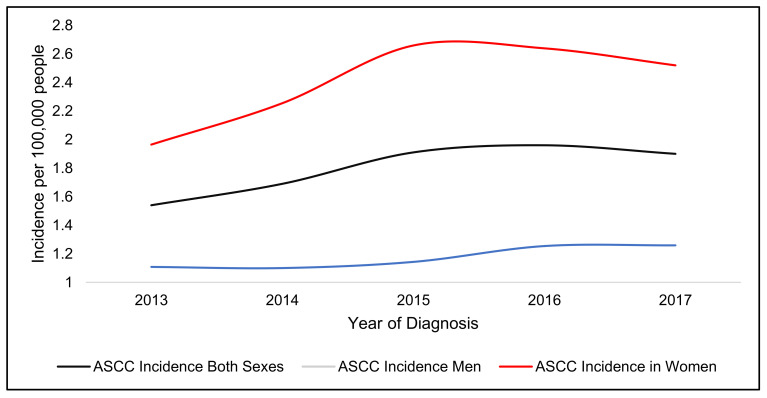
Incidence (per 100,000 people) of Anal SCC in England from 2013–2017 and gender.

Women had the highest rate of incidence (2.52 per 100,000 women) and a higher incidence increase over five years (28.6%) when compared to men (1.26 per 100,000 men and 13.5% increase over five years) (Figure 1).

**Figure 2:**
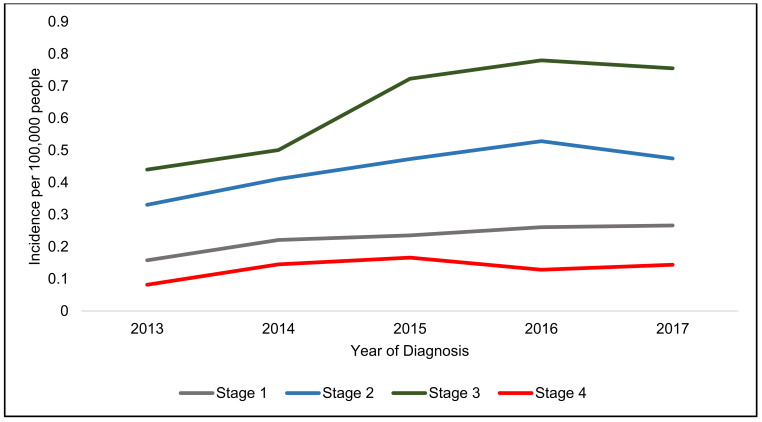
Incidence per 100,000 people in England from 2013–2017 in men and women stratified by stage.

**Figure 3:**
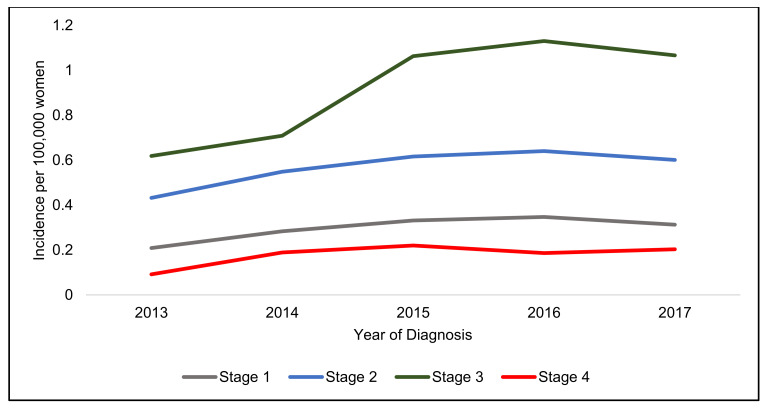
Incidence per 100,000 women in England from 2013–2017 stratified by stage.

**Figure 4:**
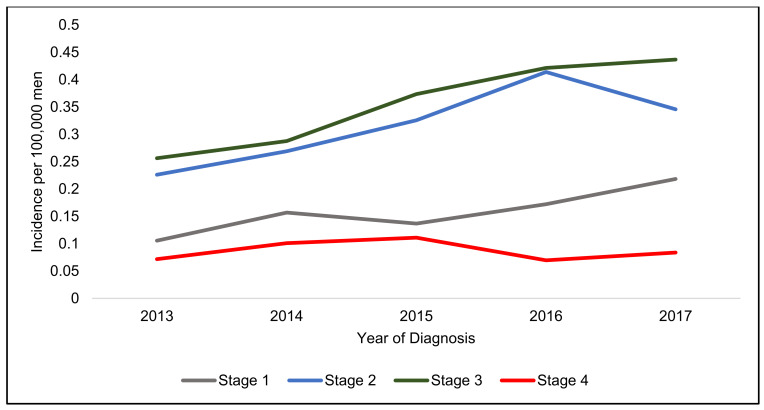
Incidence per 100,000 men in England from 2013–2017 stratified by stage.

Stage 3 disease had the highest incidence and Stage 4 had the lowest increase in both genders (Figure 2, Figure 3 and Figure 4).

There appears to be two different patterns in incidence increase, male patients have a rapidly increasing incidence of Stage 1 cancers (Table 3), whereas female patients have a similar rapid increase in Stage 4 tumours over the five years.

## 5. Discussion

Despite emphasis in the literature regarding the increasing risk of male patients living with HIV (PLWH) developing ASCC, the predominant patient group in England diagnosed with ASCC are women with Stage 3 cancers. Women also have the highest ASCC incidence and greatest incidence increase.

There has been a large increase in women presenting with metastatic stage 4 cancers and, as 45.6% of women presented with either a Stage 3 or Stage 4 tumours, there is certainly room for improvement in the early detection of ASCC in women. This is important, as the survival rates of ASCC for early stage ASCC are very good (86% Stage 1 and 77% Stage 2) [13] and 81% of T1 and T2 tumours are recurrence and progression free 3 years after treatment [1]. Currently, little research is being undertaken to improve the early detection of women with ASCC as screening programmes for ASCC are usually limited to high-risk PLWH due to a lack of evidence that the screening for and treatment of AIN is beneficial [14].

There has also been a rapid increase in men diagnosed with Stage 1 ASCC, this could be related to high risk PLWH being diagnosed within screening programmes for AIN. Interestingly, Stage 1 male patients are also more likely to have surgery as the only treatment for their malignancy. However, the dataset does not describe what procedures are undertaken, since the Lower Anogenital Squamous Terminology guidelines described superficially invasive squamous cell carcinomas (SISCCA’s) as a separate histological entity that may be amenable to surgical excision alone [15], ASCC clinical guidelines have starting to recommend considering excision alone for Stage 1 anal verge tumours [16,17]. Greater numbers of male patients with Stage 1 tumours being treated with surgery alone in this dataset could be in response to this change in practice and testament to successful screening programmes in high-risk populations.

Although most patients in this dataset were Caucasian (93.15%), this is not in keeping with the latest English national census data from 2011 where 85.4% of the English population are Caucasian, 3.45% Black and 7.5% of people were Asian [18]. Despite patients with ASCC being predominately Caucasian the small proportion of patients who were Black African or Black Caribbean were more likely to be diagnosed at a younger age with more advanced staging and not receive treatment for their malignancy. The reasons behind this are likely to be complex, and like some of the issues faced in treating other HPV related malignancies in ethnic minority groups, it is possible that within the Black ethnic community there are higher rates of PLWH presenting late with ASCC or, as Black ethnicity can be associated with a higher proportion of comorbidities, patients may not be suitable for oncological treatment. There may also be cultural reasons due to stigma preventing patients accepting treatment. Indeed, despite the decreasing overall incidence of cervical cancer [19], Black women are known to have higher incidences of cervical cancer [20], have a higher cervical cancer mortality [21] and are less likely to receive treatment for CIN once it has been identified [22]. Ethnic minorities are also known to participate less in screening programmes due to a lack of awareness of their cancer risk, feelings of shame and embarrassment [23] and avoidance of stigma related to HPV related cancers [24]. In some African countries, homosexuality and related practices remain illegal and clinical treatment for diseases thought to be related to promiscuity are a taboo. These cultural effects are most apparent in older ethnic minority women who lack awareness of the availability of screening programmes and treatment.

A significant risk factor for the development of ASCC is a previous diagnosis of HPV related dysplasia or malignancy [25]. The incidence rate of ASCC is higher in patients with previous CIN than the general population (up to 63.8 per 100,000 person years compared to less than 2.4 per 100,000 person years) [25] and different studies report up to 86% prevalence of anal HPV infections in patients who also have gynaecological dysplasias [25]. The increasing incidence of female patients with ASCC may be related to successful screening programmes where CIN is identified and treated, however, the oncogenic HPV infection remains and instead of developing cervical cancers, patients develop ASCC. Interestingly, cervical cancer rates in the USA have plateaued in the same time-period as non-cervical HPV related malignancies have increased and the burden of non-cervical HPV malignancies surpassed cervical HPV malignancies for the first time in the USA in 2013 [26].

In the UK, modelling the effect on HPV vaccination programmes suggests that the deprivation gap in ethnic minority women may get larger temporarily, as they are less likely to attend screening or receive HPV vaccination, but then over time non-minority groups will achieve herd immunity, thus benefiting ethnic minority groups [27]. It is possible that this effect is being seen in patients with ASCC, where minority groups have a temporary disproportionately poorer prognosis.

The UK Government Indices of Deprivation produces a deprivation score based on income levels, education attainment, risk of crime, employment levels, barriers to housing and services, quality of living environment and the risk of poor health and quality of life [12]. Factors associated with low SES such as; difficulties accessing primary care, low education attainment inhibiting patient compliance or limiting patient understanding of cancer risk [28], unstable employment preventing attendance at clinical appointments [29] and volatile housing leading to delays in patients being contacted after abnormal test results all have the potential to limit access to healthcare for patients with a low SES. This dataset demonstrated that patients with a lower SES were more likely to present at a younger age with ASCC, Additionally, higher levels of social deprivation were associated with requiring all three treatment modalities (surgery, chemotherapy and radiotherapy) for Stage 3 patients in this dataset. Although we are limited by the nature of our data, it is possible that we are seeing patients with higher levels of social deprivation with a poorer outcome as they require salvage surgery or stoma formation whereas Stage 3 patients with low levels of deprivation are more likely to have chemoradiotherapy alone.

Overall, ASCC incidence is rapidly increasing in England in patterns consistent with two separate populations; one male and likely related to HIV prevalence, the other female and likely related to the effect of social deprivation and ethnicity in preventing the early diagnosis of HPV related dysplasias and malignancies as well as the successful treatment of cervical malignancies resulting a reduction of cervical cancer deaths and the long term development of other perineal HPV related pathology.

### Strengths and Limitations

Despite the limitations of COSD, this study reports a large dataset of ASCC in England from 2013–2018 with a particular emphasis on the demographics, ethnicity and social deprivation of patients diagnosed with ASCC.

Our findings are limited by the data available in the COSD dataset. In particular, COSD in this form does not permit the inclusion of end outcomes such as survival or recurrence. It is possible that despite certain patient groups having a higher or lower incidence of prognostically poor staging; this may not be reflected in survival or recurrence statistics.

Additionally, the dataset does not go into full details about which treatment each patient had and is limited to whether a patient did or did not have surgery, chemotherapy or radiotherapy. Under these criteria, an abdominoperineal resection, formation of stoma and a simple examination under anaesthetic and excision biopsy would all be classified as surgery. We are also unable to describe the order in which treatment occurred and the regimes of chemoradiotherapy patients received.

The dataset is formed from anonymised data inputted by different NHS trusts and Clinical Commissioning Groups; each will have different arrangements for how these data is collated and which employees are responsible for inputting their trust’s data. If their data are inputted retrospectively, there is a potential for recall bias to exist. Although Public Health England reports that the data included in COSD are accurate, a significant limitation to using COSD for analysis is that independent researchers are not able to authenticate the data presented.

Patients are listed by their registered location at an NHS Hospital and Clinical Commissioning Group. As the dataset is anonymised, we cannot rule out duplicate entries in the dataset. The authors did their upmost to prevent the duplication of data by stratifying patients by NHS Hospital and Clinical Commissioning Groups to identify patients that could potentially be duplicate entries in the same geographical area. Although it is unlikely, it is difficult to rule out duplicate entries in multiple geographical areas (for example, if a patient moved to a different address in different geographical area of England during their treatment for ASCC).

There are still many unanswered questions regarding the best management for ASCC. As it is a rare cancer, the evidence base behind ASCC research is limited by small sample sizes. Datasets like COSD are useful in identifying demographic outcomes related to ASCC, but without end outcomes or further information involving risk factors it is difficult to draw complete conclusions. The mASCARA registry was launched in 2019 and is the first international research registry for ASCC and high-grade AIN with a novel approach combining HIV, sexual health, AIN screening and HPV related outcomes into one dataset. It also includes long term outcomes and quality of life measures for patients treated with ASCC [30]. Hopefully, this collaborative approach will best answer questions regarding ASCC and AIN demographics, screening and treatment in the future.

## 6. Conclusions

Most patients diagnosed with ASCC in England were female with Stage 3 disease.

The incidence rate of ASCC is increasing, female incidence at a faster rate than the male incidence. Female patients have an increasing incidence of late-stage tumours, whereas male patients have seen the fastest rate of increase in Stage 1 tumours. Male patients were more likely to have early staging overall (*p* < 0.001).

Patients with ASCC in England were predominately Caucasian; however, identifying as Black African or Black Caribbean is associated with presenting at a younger age with late staging (*p* < 0.001) and not receiving treatment for their malignancy (*p* < 0.001).

Patients with high social deprivation presented at an earlier age but this did not affect staging, patients with low SES were more likely to have surgery and radiotherapy (*p* = 0.041) and less likely to have chemoradiotherapy (*p* = 0.023).

## Figures and Tables

**Table 1:** Demographics of patients diagnosed with ASCC between January 2013 and June 2018 stratified by gender.

	Female (*n* = 3692)	Male (*n* = 1765)	Statistical Association
**Age**	Median = 60–64 years	Median = 60–64 years	*p* = 0.21
	Mode = 60–64 years	Mode = 65–69 years	
**Ethnicity**			*p* = 0.39
Black African/Black Caribbean	45 (1.2%)	19 (1.1%)	
Caucasian	3439 (93.2%)	1638 (92.8%)	
Indian Subcontinent	21 (0.6%)	15 (0.9%)	
Mixed Black/White	6 (0.2%)	1 (0.1%)	
Mixed Asian/White	5 (0.1%)	1 (0.1%)	
Other Mixed	3 (0.1%)	5 (0.3%)	
Chinese	2 (0.1%)	2 (0.1%)	
Other Asian	4 (0.1%)	4 (0.2%)	
Other Ethnicity	32 (0.9%)	21 (1.2%)	
Unknown Ethnicity	129 (3.5%)	59 (3.3%)	
**Deprivation score**			*p* = 0.06
Score 1 (least deprived)	605 (16.4%)	260 (14.7%)	
Score 2	735 (19.9%)	324 (18.4%)	
Score 3	719 (19.5%)	364 (20.6%)	
Score 4	791 (21.4%)	356 (20.2%)	
Score 5 (most deprived)	739 (20.0%)	403 (22.8%)	
Unknown	103 (2.8%)	58 (3.3%)	
**Staging**			*p* < 0.001
Stage 1	456 (12.4%)	228 (12.9%)	
Stage 2	875 (23.7%)	479 (27.1%)	
Stage 3	1411 (38.2%)	552 (31.3%)	
Stage 4	274 (7.4%)	135 (7.7%)	
Unknown Staging	676 (18.3%)	371 (21.0%)	

**Table 2:** Treatment modality received stratified by stage of patient at diagnosis.

Treatment Received	Stage 1 (*n* = 684)	Stage 2 (*n* = 1354)	Stage 3 (*n* = 1963)	Stage 4 (*n* = 409)	Unknown Staging (*n* = 1047)	Statistical Association
**Surgery alone**	239 (34.9%)	160 (11.8%)	127 (6.5%)	43(10.5%)	238(22.7%)	*p* < 0.001
**Chemotherapy alone**	10 (1.5%)	18 (1.3%)	45 (2.3%)	32 (7.8%)	26 (2.5%)	*p* < 0.001
**Radiotherapy alone**	42 (6.1%)	121 (8.9%)	183 (9.3%)	52 (12.7%)	105 (10.0%)	*p* = 0.01
**Surgery and Chemotherapy**	12 (1.8%)	11 (0.8%)	22 (1.1%)	19 (4.7%)	14 (1.3%)	*p* < 0.001
**Surgery and Radiotherapy**	23 (3.4%)	60 (4.4%)	84 (4.3%)	20 (4.9%)	41 (3.9%)	*p* = 0.72
**Chemoradiotherapy**	165 (24.1%)	615 (45.4%)	872 (44.4%)	97 (2.2%)	274 (26.2%)	*p* < 0.001
**Surgery and Chemoradiotherapy**	117 (17.1%)	209 (15.4%)	350 (17.8%)	49 (12.0%)	101 (9.7%)	*p* < 0.001
**No treatment**	76 (11.1%)	160 (11.8%)	280 (14.3%)	97 (23.7%)	248 (23.7%)	*p* < 0.001

**Table 3:** Percentage increase in incidence rates in England between 2013 and 2017 by gender and staging.

Staging	Incidence Rate 2017 (100,000 People)	Incidence Increase (%)	Incidence Rate 2017 (100,000 Women)	Incidence Increase (%)	Incidence Rate 2017 (100,000 Men)	Incidence Increase (%)
**Stage 1**	0.27	68.6	0.31	50.0	0.22	106.9
**Stage 2**	0.47	43.6	0.60	39.1	0.35	52.9
**Stage 3**	0.76	71.6	1.07	72.4	0.44	70.4
**Stage 4**	0.14	76.1	0.20	121.5	0.08	16.9

## Data Availability

The complete datasets generated and analysed in this study are available to healthcare workers from the Cancer Outcomes Services Dataset maintained by Public Health England.

## References

[1] James R.D., Glynne-Jones R., Meadows H.M., Cunningham D., Myint A.S., Saunders M.P., Maughan T., McDonald A., Essapen S., Leslie M. (2013). Mitomycin or cisplatin chemoradiation with or without maintenance chemotherapy for treatment of squamous-cell carcinoma of the anus (ACT II): A randomised, phase 3, open-label, 2×2 factorial trial. Lancet Oncol..

[2] Islami F., Ferlay J., Lortet-Tieulent J., Bray F., Jemal A. (2017). International trends in anal cancer incidence rates. Int. J. Epidemiol..

[3] Tweed E.J., Allardice G.M., McLoone P., Morrison D.S. (2018). Socio-economic inequalities in the incidence of four common cancers: A population-based registry study. Public Health.

[4] Teng A.M., Atkinson J., Disney G., Wilson N., Blakely T. (2017). Changing socioeconomic inequalities in cancer incidence and mortality: Cohort study with 54 million person-years follow-up 1981-2011. Int. J. Cancer.

[5] Woods L.M., Rachet B., Coleman M.P. (2006). Origins of socio-economic inequalities in cancer survival: A review. Annals Oncol. Off. J. Eur. Soc. Med. Oncol..

[6] Lyratzopoulos G., Abel G.A., Brown C.H., Rous B.A., Vernon S.A., Roland M., Greenberg D.C. (2013). Socio-demographic inequalities in stage of cancer diagnosis: Evidence from patients with female breast, lung, colon, rectal, prostate, renal, bladder, melanoma, ovarian and endometrial cancer. Annals Oncol. Off. J. Eur. Soc. Med. Oncol..

[7] Bryere J., Dejardin O., Launay L., Colonna M., Grosclaude P., Launoy G., French Network of Cancer R. (2018). Socioeconomic status and site-specific cancer incidence, a Bayesian approach in a French Cancer Registries Network study. Eur. J. Cancer Prev. Off. J. Eur. Cancer Prev. Organ. (ECP).

[8] Celie K.-B., Jackson C., Agrawal S., Dodhia C., Guzman C., Kaufman T., Hellenthal N., Monie D., Monzon J., Oceguera L. (2017). Socioeconomic and gender disparities in anal cancer diagnosis and treatment. Surg. Oncol..

[9] Lin D., Gold H.T., Schreiber D., Leichman L.P., Sherman S.E., Becker D.J. (2018). Impact of socioeconomic status on survival for patients with anal cancer. Cancer.

[10] Ahmad T.R., Susko M., Lindquist K., Anwar M. (2019). Socioeconomic disparities in timeliness of care and outcomes for anal cancer patients. Cancer Med..

[11] von Elm E., Altman D.G., Egger M., Pocock S.J., Gøtzsche P.C., Vandenbroucke J.P., STROBE Initiative (2007). The Strengthening the Reporting of Observational Studies in Epidemiology (STROBE) Statement: Guidelines for Reporting Observational Studies. PLoS Med..

[12] Ministry of Housing, Communities and Local Government English Indices of Deprivation. https://www.gov.uk/government/statistics/english-indices-of-deprivation-2019.

[13] Goffredo P., Utria A., Engelbart J., Masson A., Kalakoti P., Hassan I. (2018). The impact of patient demographics vs tumor factors on the prognosis of anal squamous cell carcinoma treated with standard chemoradiation therapy. Dis. Colon Rectum.

[14] Brogden D.R.L., Walsh U., Pellino G., Kontovounisios C., Tekkis P., Mills S.C. (2021). Evaluating the efficacy of treatment options for anal intraepithelial neoplasia: A systematic review. Int. J. Colorectal Dis..

[15] Darragh T.M., Colgan T.J., Thomas Cox J., Heller D.S., Henry M.R., Luff R.D., McCalmont T., Nayar R., Palefsky J.M., Stoler M.H. (2013). The Lower Anogenital Squamous Terminology Standardization project for HPV-associated lesions: Background and consensus recommendations from the College of American Pathologists and the American Society for Colposcopy and Cervical Pathology. Int. J. Gynecol. Pathol..

[16] Geh I., Gollins S., Renehan A., Scholefield J., Goh V., Prezzi D., Moran B., Bower M., Alfa-Wali M., Adams R. (2017). Association of Coloproctology of Great Britain & Ireland (ACPGBI): Guidelines for the Management of Cancer of the Colon, Rectum and Anus (2017)—Anal Cancer. Colorectal Dis..

[17] Stewart D.B., Gaertner W.B., Glasgow S.C., Herzig D.O., Feingold D., Steele S.R., Rectal S., Prepared on Behalf of the Clinical Practice Guidelines Committee of the American Society of Colon and Rectal Surgeons (2018). The American Society of Colon and Rectal Surgeons Clinical Practice Guidelines for Anal Squamous Cell Cancers (Revised 2018). Dis. Colon Rectum.

[18] Office for National Statistics (2011). National Census. https://www.ons.gov.uk/census/2011census/2011censusdata.

[19] Islami F., Fedewa S.A., Jemal A. (2019). Trends in cervical cancer incidence rates by age, race/ethnicity, histological subtype, and stage at diagnosis in the United States. Prev. Med..

[20] Clegg L.X., Reichman M.E., Miller B.A., Hankey B.F., Singh G.K., Lin Y.D., Goodman M.T., Lynch C.F., Schwartz S.M., Chen V.W. (2009). Impact of socioeconomic status on cancer incidence and stage at diagnosis: Selected findings from the surveillance, epidemiology, and end results: National Longitudinal Mortality Study. Cancer Causes Control CCC.

[21] Beavis A.L., Gravitt P.E., Rositch A.F. (2017). Hysterectomy-corrected cervical cancer mortality rates reveal a larger racial disparity in the United States. Cancer.

[22] Akers A.Y., Newmann S.J., Smith J.S. (2007). Factors underlying disparities in cervical cancer incidence, screening, and treatment in the United States. Curr. Probl. Cancer.

[23] Marlow L.A.V., Waller J., Wardle J. (2015). Barriers to cervical cancer screening among ethnic minority women: A qualitative study. J. Fam. Plan.Reprod. Health Care.

[24] Vrinten C., Gallagher A., Waller J., Marlow L.A.V. (2019). Cancer stigma and cancer screening attendance: A population based survey in England. BMC Cancer.

[25] Stier E.A., Sebring M.C., Mendez A.E., Ba F.S., Trimble D.D., Chiao E.Y. (2015). Prevalence of anal human papillomavirus infection and anal HPV-related disorders in women: A systematic review. Am. J. Obstet. Gynecol..

[26] Deshmukh A.A., Suk R., Shiels M.S., Damgacioglu H., Lin Y.Y., Stier E.A., Nyitray A.G., Chiao E.Y., Nemutlu G.S., Chhatwal J. (2021). Incidence trends and burden of human papillomavirus-associated cancers among women in the United States, 2001–2017. J. Natl. Cancer Inst..

[27] Johnson H.C., Lafferty E.I., Eggo R.M., Louie K., Soldan K., Waller J., Edmunds W.J. (2018). Effect of HPV vaccination and cervical cancer screening in England by ethnicity: A modelling study. Lancet Public Health.

[28] Murfin J., Irvine F., Meechan-Rogers R., Swift A. (2020). Education, income and occupation and their influence on the uptake of cervical cancer prevention strategies: A systematic review. J. Clin. Nurs..

[29] Costas-Muniz R., Leng J., Aragones A., Ramirez J., Roberts N., Mujawar M.I., Gany F. (2016). Association of socioeconomic and practical unmet needs with self-reported nonadherence to cancer treatment appointments in low-income Latino and Black cancer patients. Ethn. Health.

[30] Brogden D.R.L., Kontovounisios C., Pellino G., Bower M., Mills S.C., Tekkis P.P. (2019). Improving outcomes for the treatment of Anal Squamous Cell Carcinoma and Anal Intraepithelial Neoplasia. Tech. Coloproctol..

